# Formulation and Quality Assessment of a Functional *Foléré* Beverage Enriched With Tamarind Pulp

**DOI:** 10.1155/ijfo/8885380

**Published:** 2025-09-03

**Authors:** Yaya Bruno Foundikou, Darman Roger Djoulde, Harouna Difo Voukang, Daoudou Bakari, Yonas Vandi

**Affiliations:** ^1^ Department of Biological Sciences, Faculty of Science, University of Maroua, Maroua, Cameroon, uni-maroua.citi.cm; ^2^ Department of Agriculture, Livestock and Derived Products, National Advanced School of Engineering Maroua, University of Maroua, Maroua, Cameroon, uni-maroua.citi.cm

**Keywords:** *foléré*, functional beverage, hibiscus calyx, quality, tamarind fruit

## Abstract

*Foléré* is a traditional Cameroonian non‐alcoholic beverage derived from the calyx of *Hibiscus sabdariffa.* This study evaluated the nutritional, bioactive, physicochemical and microbiological properties of *foléré* enriched with tamarind (*Tamarindus indica*) fruit pulp. The beverage was prepared by boiling hibiscus calyces (1:4 w/v), sweetening with 16% sucrose and enriching with 20 mg/mL tamarind extract followed by pasteurisation (65°C/30 min). The resulting red‐pigmented drink had a pH of 2.52, 0.54% total acidity, 13.2°Brix total soluble solids, 1545.67 ppm total dissolved solids and 3092 *μ*S/cm electrical conductivity. Despite the high moisture content (82.63%), it contained appreciable levels of reducing sugars (32.42 mg/L), proteins (0.95 mg/L) and carbohydrates (50.7 mg/L). Bioactive compounds included flavonoids (307.02 mg/L), polyphenols (496.11 mg/L), tannins (366.12 mg/L), ascorbic acid (6.5 mg/100 mL) and anthocyanins (68.22 mg/L). Antioxidant activity was notable with 42.88% DPPH inhibition and 654.58 mg/L FRA*P* value. Microbiological analysis confirmed an acceptable hygienic quality. These findings highlight the potential of tamarind‐enriched *foléré* as a nutritious, antioxidant‐rich alternative to imported beverages. Further studies are recommended to assess the shelf life and long‐term safety.

## 1. Introduction

Functional beverages are non‐alcoholic bioactive drinks taken not only to quench thirst but also to refill nutritional deficits and relieve bodily activities [1]. They are generally made from plants, animals or marine organisms [1]. Fruit juices and fruit nectars are the most popular functional drinks after dairy beverages and tea. In the last decades, health wellness is the major concern of consumers and the food industry as a whole. This change of habit has perpetuated the demand of fruit juices and fruit nectars with a market size that stood at $132.31 billion in 2023 and is expected to reach $186.01 billion by 2032 [2]. Unfortunately, the Middle East and Africa are regions with the lowest production and consumption rate of fruit juices and nectars behind Europe [3]. These countries rely on the importation of exotic fruit juices and fruit nectars, which often are costly to the greater fraction of the population that lives beyond 2 US dollar as is the case in Cameroon [4]. This therefore reduces their ability to afford a sip of these beverages. Fortunately, Africa has a multifarious spectrum of fruit trees. In Africa, fruits are mostly seasonal, and the vast majority decompose faster if not preserved. Alternatively, the *Hibiscus sabdariffa* calyx is constantly available because it can be dried and stored for years without alteration. This reduces the bulkiness and facilitates the transportation and distribution of hibiscus calyx in different markets across the world.

In Africa, the hibiscus calyx is broadly used to prepare a non‐alcoholic beverage called *foléré* in Cameroon, *karkade* in Egypt, *zobo* in Nigeria, *zobolo* in Ghana, *bissap* in Guinea and Ivory Coast, sorrel/roselle in English, *jus d’oseille* in French and Jamaican sorrel in Jamaica [5, 6]. This beverage is taken as either a tea or a refreshing drink without gender discrimination or religious beliefs [5]. The production and commercialisation of *foléré* is vibrant in Cameroon and often skips the attention of regulatory institutions. According to records, Bayoï et al. [7] described the manufacturing process of *foléré* and reported that the poor mastering of the manufacturing processes and unhygienic environment affected the quality of *foléré* drink. The need to improve the technological production and quality of *foléré* is a fact especially in a country like Cameroon.

Functional beverages are mostly exempt from chemical preservatives and rely solemnly on the use of physical and biological techniques of preservation to achieve a stable quality. Biological techniques involving the use of edible plant extracts are less costly and often characterised by additive qualities to the product rather than physical techniques. In West Africa, it has been reported that the use of edible plant extracts have improved the quality of *foléré* or related drinks [8, 9]. Consequently, the use of spices/edible plants remains a vital alternative needed to enhance the quality of *foléré* drink.

Tamarind or *Tamarindus indica* is a wild plant of Cameroon, Sudan, Nigeria, Zambia and Tanzania whose fruit is repeatedly used in culinary activities and folk medicine [10]. It has been reported by Bayoï et al. [11] that the leaves of tamarind could improve the sensory quality as well as the shelf life of *foléré*. Like with Foundikou et al. [12], the studies undertaken by Bayoï et al. [7, 11] did not account for the nutritional, bioactive and microbiological properties of *foléré*. Though the fruit pulp of tamarind is highly prized and nutritive, its extracts have revealed the presence of bioactive compounds with a spectrum of actions against the agents of food intoxication [13, 14]. Hence, the fruit pulp of tamarind can be used as a preservative. As such, the blend between hibiscus calyx and tamarind fruit pulp could stand as an alternative to uplift the quality of *foléré* beverage. Therefore, this study evaluated the nutritional, bioactive and microbiological qualities of *foléré* enriched with tamarind fruit pulp extract.

## 2. Materials and Methods

### 2.1. Collection and Preparation of Plant Materials

The freshly mature fruits of *Tamarindus indica* were harvested during the early month of November 2021 from Daguisan Markaba village, Meri subdivision, Diamaré division, Far North Region, Cameroon, located between latitude 14° 13′ 12″ E and longitude 10° 48′ 0″ N. The harvested fruit was transported in a polythene bag to the Biological Sciences Laboratory, Faculty of Science, University of Maroua. The fresh fruits were shade‐dried at room temperature until a constant weight was attained, manually deseeded, and the pulp was ground using a mechanical hand blender (Victoria, China). The fruit was identified at the National Herbarium of Cameroon under the Code YA0002274/1964. The fruit pulp was macerated in the extracting solvent [15]. Twenty‐five grams (25 g) of the fruit was soaked in 150 mL hydroethanolic solution (70%) in an Erlenmeyer flask. The mixture was homogenised using a magnetic stirrer after every 20 min for a period of 2 h to completely dissolve the ground fruit. The mixture was filtered (Whatman No. 4) after 24 h of maceration. The resulting filtrate was concentrated by drying at reduced temperature (50°C) and pressure in a vacuum air oven (Memmert GmbH + Co. KG, Germany).

On the other hand, the reddish purple dried calyx of *Hibiscus sabdariffa* was purchased from wholesalers of the Maroua II Municipal market. The calyx was identified at the National Herbarium of Cameroon under the Code YA0051542/1977. The calyx was sorted to eliminate the dirt. The calyx was mechanically ground and sieved with a 200 *μ*m diameter sieve to obtain a powder which was stored in a black sterile polythene bag at ambient temperature (30 ± 2^°^C) until used.

### 2.2. Manufacturing Process of Foléré Beverage

**Figure 1:**
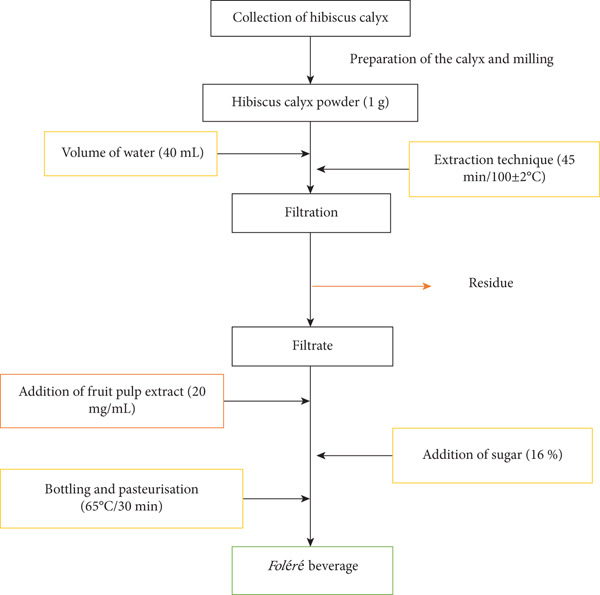
Manufacturing layout of *foléré* beverage.

The beverage was prepared according to the procedure described by Foundikou et al. [12]. This procedure is illustrated in Figure 1. Summarily, the dried hibiscus calyx was manually sorted to discard unwanted particles and rotten calyx. The calyx was ground using a mechanical blender and sieved to obtain a homogeneous powder. The ground hibiscus calyx and distilled water were mixed in the ratio 1:40 (w/v) and boiled for 45 min at 100 ± 2^°^C. The mixture was allowed to cool and filtered using a sieve (200 *μ*m). The volume of the filtrate was completed to the initial volume with sterile distilled water, sweetened with granulated refined sucrose (16%) and enriched with 20 mg/mL tamarind fruit pulp extract. The mixture was vigorously stirred for 15 min and transferred into sterile recycled transparent glass bottles, corked and pasteurised for 30 min at 65°C. The beverage was rapidly cooled by immersing it in fresh cold water.

### 2.3. Physicochemical Properties

The electrical conductivity (microsiemens per centimetre), total dissolved solids (parts per million), total soluble solids (°Brix) and pH were determined using portable devices. A multifunctional portable conductivity metre type (e‐1 TDS & EC) was used to take the readings for the electrical conductivity and total dissolved solids; meanwhile, the pH metre (Eco Testr, Singapore) and ATC refractometer (RHB 90, Shenzhen, China) were used to measure the pH and total soluble solids. The values of the pH, electrical conductivity, total dissolved solids and total soluble solids were directly measured by immersing the corresponding apparatus in 10 mL beverage sample after calibration. The total titratable acidity (TA) was assayed according to the approach described by Ramirez et al. [16], and the result was expressed in % of malic acid equivalent (MAE) per millilitre.

### 2.4. Proximate Analysis

#### 2.4.1. Ash Content

The ash content was determined according to the method described by Nielsen [17]. One hundred millilitres (100 mL) of the aliquot was weighed into a tared crucible and placed in a cool muffle furnace. The muffle furnace (FNC‐BX1200 Series, Infitek, Shanghai, China) was ignited for 12–18 h (or overnight) at 550°C. The furnace was turned off and allowed to cool. The ash content was calculated, and the result was expressed in %:

(1)  Ash content %=wt after ashing−tare wt of crucibleoriginal sample wt ×100

where wt shows the weight in grammes.

#### 2.4.2. Moisture Content

The moisture content was determined according to the method described by Ukwo et al. [18]. The crucible was weighed, and the mass, *M*_1_, was recorded. Ten millilitres (10 mL) of the sample was measured in a clean crucible, and the mass, *M*_2_, was noted. The crucible was placed in an air‐dry oven (Memmert GmbH + Co. KG, Germany) set at 105°C for 3 h, after which the mass, *M*_3_, was recorded. The crucible was reinserted into the oven (Memmert GmbH + Co. KG, Germany) and weighed after every 30 min until the constant weight was reached. The moisture content was calculated, and the result was expressed in % [19]:

(2) Moisture content %=M2−M1−M3−M1M2−M1×100

where

*M*_1_ = weight of empty crucible.

*M*_2_ = weight of crucible + sample.

*M*_3_ = weight of crucible + dry sample.

#### 2.4.3. Total Protein Content

The total protein content was determined using the Bradford dye binding method [20]. One millilitre (1 mL) of the sample or bovine serum albumin (BSA) solution (0–100 *μ*g/mL) was pipetted, and 5 mL of Bradford reagent (Coomassie Brilliant Blue G‐250 is dissolved in 95% ethanol and acidified with 85% phosphoric acid) was carefully added. The content was incubated for 5 min, and the absorbance was read at 595 nm against a blank prepared under experimental conditions with distilled water. The total protein content was extrapolated from the BSA standard curve, and the result was expressed in milligram BSA equivalent per litre of beverage.

#### 2.4.4. Total Reducing Sugar

The total reducing sugar content was colourimetrically determined using 3,5‐dinitrosalicylic acid (DNSA) reagent [21]. One hundred microlitres (100 *μ*L) of the sample or galactose solution (0–100 *μ*g/mL) was introduced into test tubes, followed by the addition of 1 mL DNSA solution, and the mixture was homogenised. The content was heated in a hot water bath at 95°C for 5 min. After cooling, 1 mL of distilled water was added, and the absorbances were read at 540 nm against the blank tube prepared under the same experimental conditions. The total reducing sugar content was extrapolated from the galactose standard curve, and the result was expressed in milligrams galactose equivalent per litre of the beverage.

#### 2.4.5. Total Carbohydrate Content

The phenol–sulphuric acid method described by Nielsen [17] was used to determine the total carbohydrate content. One hundred microlitres (100 *μ*L) of the beverage sample or galactose solution (0–100 *μ*g/mL) together with 1 mL 5% phenol solution was mixed and shaken. This was followed by the careful addition of 5 mL 96% H_2_SO_4_. The mixture was carefully homogenised for 2 min and heated in a hot water bath at 95°C for 30 min. The mixture was allowed to cool, and the absorbance of the golden yellow colour solution of the sample was read at 490 nm against the blank prepared with distilled water under the same experimental conditions. The total carbohydrate content was extrapolated from the galactose standard curve, and the result was expressed in milligram galactose equivalent per litre of the beverage.

### 2.5. Phytochemical Content of Foléré

#### 2.5.1. Total Polyphenol Content (TPC)

The concentration of polyphenols was determined spectrophotometrically using the Folin–Ciocalteu method [22]. Two hundred microlitres (200 *μ*L) of the beverage or gallic acid standard solution (0–250 *μ*g/mL) was mixed with 1 mL of Folin–Ciocalteu reagents (1:10 v/v) and 1 mL of 7.5% Na_2_CO_3_ (m/v) solution. The mixture was homogenised and incubated for 70 min at room temperature, and the absorbance at 765 nm was read. The concentration of polyphenol was extrapolated from the gallic acid standard curve, and the result was expressed in milligrams of gallic acid equivalent per litre of the beverage.

#### 2.5.2. Total Flavonoid Content (TFC)

The aluminium chloride (AlCl_3_) colorimetric method was used to evaluate the TFC [23]. Two hundred microlitres (200 *μ*L) of the beverage sample or quercetin standard solution (0–250 *μ*g/mL) was mixed with 1.9 mL distilled water and 0.15 mL of 5% NaNO_2_. The mixture was incubated for 5 min at ambient temperature, after which 0.15 mL of 10% AlCl_3_ and 0.5 mL 1 mM NaOH were added, homogenised and incubated for 25 min. The absorbance was measured at 510 nm, and the TFC was expressed in milligrams of quercetin equivalent per litre of the beverage after extrapolation for the standard curve established with quercetin.

#### 2.5.3. Total Condensed Tannin Content (TTC)

The TTC was determined using the vanillin acid method [24]. Two hundred microlitres (200 *μ*L) beverage sample or catechin standard solution (0–250 *μ*g/mL) was mixed with 1.5 mL of vanillin acid, and the whole mixture was homogenised and incubated for 20 min at room temperature. The absorbance was read at 500 nm, and the TTC was expressed in milligrams of catechin per litre of the beverage.

### 2.6. Antioxidant Potentials of Foléré

#### 2.6.1. 2,2‐Diphenyl‐1‐Picrylhydrazyl (DPPH) Free Radical Scavenging Activity

The DPPH scavenging activity was determined using the stable radical DPPH photometric assay as a reagent [25]. Two hundred microlitres (200 *μ*L) of beverage sample was mixed with 1.5 mL of ethanolic DPPH solution, and the absorbance at 517 nm was measured after 30 min of incubation in darkness. The DPPH free radical scavenging activity of the beverage was calculated using the formula below [26] and expressed as a percentage of inhibition (I %):

(3) I%=ODcontrol−ODsampleODcontrol×100

where

*O* *D*_*s* *a* *m* *p* *l* *e*_: absorbance of the DPPH solution containing samples.

*O* *D*_*c* *o* *n* *t* *r* *o* *l*_: absorbance of the control solution without sample but with DPPH.

*I* *%*: inhibition percentage of DPPH pro‐oxidant activity.

#### 2.6.2. Ferric Reducing Antioxidant Power (FRAP) Assay

The FRAP was evaluated according to the method described by Bibi Sadeer et al. [22]. Two hundred microlitres (200 *μ*L) of the beverage or Trolox solution (0–250 *μ*g/mL) was mixed with 1.5 mL of ferric‐tripyridyltriazine (Fe[TPTZ]_2_)^3+^ solution, homogenised and incubated for 30 min in darkness. The optical density was measured at 593 nm. The FRAP content was extrapolated from the Trolox standard graph, and the result was expressed as milligrams of Trolox equivalent per litre of the beverage.

#### 2.6.3. Ascorbic Acid Content

The vitamin C content was determined using the dichlorophenolindophenol reagent [27]. Summarily, 1 mL of the beverage was mixed with 9 mL of metaphosphoric acid–acetic acid solution. The solution was homogenised for 5 min, and 1 mL of the vortexed solution or l‐ascorbic acid standard solution (0–80 *μ*g/mL) was mixed with 1 mL of 2,6‐dichlorophenolindophenol and homogenised. The absorbance of the mixture was directly taken in a UV–Vis spectrophotometer (Jenway 7305, Bibby Scientific, Group HQ, United Kingdom) at 520 nm. The ascorbic acid content was extrapolated from the standard curve prepared with l‐ascorbic acid, and the result was expressed as milligrams of l‐ascorbic acid equivalent (AAE) per 100 mL (mg AAE/100 mL) of the beverage.

### 2.7. Total Monomeric Anthocyanin Content (TAC)

The total anthocyanin content was determined using the pH differential method [28]. One millilitre of the beverage was mixed with 9 mL acidified ethanolic solution and allowed to stand for 1 h and filtered (Whatman No. 4). Two hundred microlitres (200 *μ*L) of the filtrate or acidified ethanolic solution of cyanidin‐3‐glucoside (50–250 *μ*g/mL) was pipetted into test tubes in triplicates. The pH was adjusted to 1 and 4.5 with 1.8 mL of KCl buffer solution (0.025 M) and sodium acetate buffer solution (0.4 M), respectively. The absorbances were read against the blank prepared under the same experimental conditions using acidified ethanolic solution at 520 and 700 nm after 20–50 min of incubation. The total anthocyanin content was calculated [28], and the result was expressed as milligrams of cyanidin‐3‐glucoside equivalent per litre of *foléré* beverage:

(4) Total anthocyanins mg/L=A×MW×DF×103ε×l

where

*A* = (*A*_520_ − *A*_700_) *p* *H*_1_ − (*A*_520_ − *A*_700_) *p* *H*_4.5_.

*A*_520_ = optical density at 520 nm.

*A*_700_ = optical density at 700 nm.

MW = molecular weight of cyanidin‐3‐glucoside (449.2 g/mol).

DF = dilution factor.

*ε* = molar extinction coefficient (26,900 L/mol/cm for cyanidin‐3‐glucoside).

*l* = path length (1 cm).

### 2.8. *α*‐Carotene, *β*‐Carotene, Lycopene and Lutein Content

The carotenoid content (*α*‐carotene, *β*‐carotene, lycopene and lutein) was determined according to the protocol described by [29]. One millilitre (1 mL) beverage sample was mixed with 2 mL of hexane/acetone (3:2). The mixture was homogenised for 1 min and allowed to settle for 5 min. The organic phase (supernatant) was pipetted and filtered (Whatman #4); meanwhile, the decant was mixed again with the same volume of solvent, and the operation was repeated. All the filtrates were combined to make one sample. The optical density of the organic phase was read at 445, 450, 470 and 442 nm to quantify the amount of *α*‐carotene, *β*‐carotene, lycopene and lutein, respectively. The total content of *α*‐carotene, *β*‐carotene, lycopene and lutein was determined, and the results were expressed accordingly [29]:

(5) α‐Carotene,β‐carotene,lycopene and lutein mg100mL=OD×V×DFWs×E1%

where OD is the optical density (nanometre), *V* is the volume of the extract (millilitre), DF is the dilution factor, Ws is the weight of the sample (milligramme) and *E* ^1%^ is the absorption coefficient of *α*‐carotene (2725 cm^−1^), *β*‐carotene (2592 cm^−1^), lycopene (3450 cm^−1^) and lutein (2550 cm^−1^).

### 2.9. Colour Intensity, Colour and Hue Tint

The colour intensity and colour of *foléré* were determined as described by Kalkan Yildirim [30]. The spectrophotometric absorbances of the beverage (0.2 mL) were read using a 1 cm cell path length at 420, 520 and 620 nm. The colour intensity (IC) was calculated as the sum of the absorbances at 420, 520 and 620 nm. The hue tint (*T*) was calculated as the ratio of *A*_420_ to *A*_520_. The proportion of red, yellow and blue colour of the beverage was calculated:

(6)  Red colour %=A520×100IC,

(7) Yellow colour %=A420×100IC,

(8) Blue colour %=A620×100IC.

### 2.10. Microbiological Analysis

The serial dilution method and surface plating technique as described by Loyer and Hamilton [31] and Sanders [32] were used to prepare the inoculum and seeding, respectively. One hundred microlitres (100 *μ*L) of serial diluted sample was surface spread in different agars to enumerate the corresponding flora. The seeded plates were allowed to stand for 10 min, inverted and incubated at optimum temperature of the flora of interest. Prior to the inoculation and enumeration of spore‐forming bacteria, the serial diluted tubes were thermally activated at 80°C for 10 min [33]. The total viable count (TVC) and total spore‐forming bacteria count were enumerated in plate count agar after incubating at 35°C for 24 h. Similarly, the total and faecal coliforms count were enumerated in eosin‐methylene blue agar after 24 h of incubation at 35°C and 44°C for total coliforms and faecal coliforms, respectively. The total fungi (mould and yeast) counts were enumerated in Sabouraud agar supplemented with chloramphenicol after 72 h of incubation at 25°C [34]. The microbial concentration of each flora was calculated, and the result was expressed in colony forming units (CFUs) per millilitre:

(9) Microbial load CFU/mL=nD×V

where

*n* = number of enumerated colonies.

*D* = dilution.

*V* = inoculated volume (millilitre).

Discrete colonies from the freshly cultured plates were isolated and cultured in nutrient broth. The DNA was extracted using the DNeasy 96 Blood & Tissue Kit. The DNA was amplified using the 16S rRNA PCR technique [35]. Summarily, PCR amplification was performed in a final reaction volume of 25 *μ*L in a PCR tube, containing 12.5 *μ*L of master mix (New England Biolabs, containing a mixture of dNTP, Taq polymerase, MgCl_2_ and PCR buffer), 2 *μ*L forward primer (5′‐AGA GTT TGA TCC TGG CTC AG‐3′), 2 *μ*L reverse primer (5′‐GGT TAC CTT CTT ACG ACT T‐3′), 2 *μ*L of extracted DNA and 6.5 *μ*L of nuclease‐free water. The thermal cycles for the amplification were set as follows: initial denaturation for 5 min at 94°C, 30 cycles of 40 s at 94°C, 30 s at 54°C and 1 min at 72°C and final extension for 5 min at 72°C. Thereafter, 5 *μ*L of each PCR product was analysed on 1% (w/v) agarose gel supplemented with ethidium bromide. The amplicons were then visualised using a gel documentation system and photographed. Furthermore, the PCR amplicons were sequenced using the Applied Biosystems (Life Technologies) ABI Ready Reaction Kit BigDye Terminator V3.1 Cycle protocol. The ABI sequence was converted to FASTA sequence and compared to other sequences available in the GenBank database of NCBI using BLASTn. The microorganisms were therefore identified on the basis of the percentage similarities.

### 2.11. Data Analyses

Each analysis was done in triplicates. The mean value and standard deviation for each set of data were determined. The results obtained were either tabulated or graphically presented as bar graphs. The bar graph was generated using MS Office 2016 (Office 2016, Microsoft Corp., Redmond, Washington) for Windows.

## 3. Results and Discussion

### 3.1. Proximate Composition

The total reducing sugar, carbohydrate, protein, ash and moisture contents of *foléré* drink are presented in Table 1. *Foléré* drink is a juicy concoction with a high moisture content (82.63%). This moisture content is less than 86%–88% registered by Gbadegesin et al. [36] with roselle extract blended or not with pineapple juice in Nigeria. A similar observation was made with roselle extracts flavoured with tropical fruits extracts in Nigeria [37]. The low moisture content registered with *foléré* drink is clear evidence that *foléré* drink produced in Cameroon is more concentrated than those from Nigeria. However, the moisture content falls within the range of 88%–79% reported by Ukwo et al. [18] with roselle extract blended with varying proportions of date fruit extract. Despite the high moisture content, the ash content of 1.06% was greater than 0.31%–0.95% recorded by Fasoyiro et al. [37] and Ukwo et al. [18] with roselle juice blended with tropical fruits and date fruit extracts, respectively. The high ash content could supposedly indicate the presence of minerals. It has been reported by Fasoyiro et al. [37] and Mgaya‐Kilima et al. [38] that *foléré*‐related beverages contain both micronutrients and trace elements. As such, the consumption of *foléré* beverage could in one way or the other relieve certain mineral deficiencies especially in children.

**Table 1:** Proximate composition of *foléré* beverage.

**Parameters**	**M** **e** **a** **n** ± **S** **D**
Moisture content (%)	82.63 ± 0.17
Ash content (%)	1.06 ± 0.09
TRS (mg/L)	32.42 ± 0.44
TCC (mg/L)	50.70 ± 0.42
TPC (mg/L)	0.95 ± 0.05

Abbreviations: SD, standard deviation; TCC, total carbohydrates content; TPC, total protein content; TRS, total reducing sugar.

Additionally, *foléré* drink is rich in reducing sugars and total carbohydrates with traces of protein. The total carbohydrate and protein contents of the current study are greater than the contents reported by Fasoyiro et al. [37] and Ukwo et al. [18] with flavoured roselle juice in Nigeria. In the same train, Bayoï et al. [7] reported a greater carbohydrate content with *foléré* juice sold in Mora, Mokolo and Diamaré, Far North Region, Cameroon. Multiple nonconventional beverages are known in the northern regions of Cameroon, but the total reducing sugar, carbohydrate and protein contents in the present study are greater than in *kounou* reported by Bayoï et al. [39] but less than in *Alme Ardeb* and *Kargasok* reported by Bakari et al. [40, 41]. The raw materials and technological processing of these indigenous beverages must have accounted for these differences. Nevertheless, *foléré* is highly nutritive, and as such, it may be considered an alternative source of nutrients to consumers.

### 3.2. Physicochemical Characteristics

The pH, total TA, conductivity, soluble solids and dissolved solids of *foléré* are summarised in Table 2. A pH of 2.52 was registered with *foléré*. This pH is less than those registered by Ukwo et al. [18] and Fasoyiro et al. [37] with roselle extract blended with edible fruits but greater than 1.89–2.12 pH values reported by Gbadegesin et al. [36] with roselle extract blended or not with pineapple juice. However, the pH was similar to that recorded by Ramirez et al. [16]. At the same time, the total TA was less than 0.96%–1.94% reported by Mgaya‐Kilima et al. [38] and Gbadegesin et al. [36] but greater than those registered by Ukwo et al. [18] with roselle beverage blended with extracts from tropical fruits in Tanzania and Nigeria. With the aforementioned properties, *foléré* drink is considered acidic and should not be consumed with an empty belly.

**Table 2:** Physicochemical properties of *foléré* beverage.

**Parameters**	**M** **e** **a** **n** ± **S** **D**
pH	2.52 ± 0.09
TA (%)	0.54 ± 0.01
TSS (°Brix)	13.2 ± 0.01
TDS (ppm)	1545.67 ± 79.22
Conductivity (*μ*S/cm)	3092 ± 158.98

Abbreviations: SD, standard deviation; TA, total titratable acidity; TDS, total dissolved solids; TSS, total soluble solids.

Besides the pH and total TA, the total soluble solids (13.2°Brix), total dissolved solids (1545.67 ppm) and electrical conductivity (3092 *μ*S/cm) were registered with *foléré*. The total soluble solids were close to 11.2°Brix–13.2°Brix registered by Bolade et al. [42], but less than 18.8°Brix–23.5°Brix registered by Gbadegesin et al. [36] with roselle extracts blended with pineapple fruit juice. Soluble solids are the measure of the concentration of soluble sugars and organic acids in beverages, fruits and vegetables. The hibiscus calyx is highly acidic; the use of sweetener to mask this acidity varies from one producer to another. Indeed, Gbadegesin et al. [36] used 20% sucrose as sweetener during the processing of roselle juice, contrary to the 16% used in the current study. Despite the close relation in the soluble solids registered by Bolade et al. [42], the differences in the amount of sweetener remained relatively important (≤ 3% of sucrose) and, above all, the calyx‐to‐water ratio and the duration of extraction.

### 3.3. Phytochemical Contents

**Figure 2:**
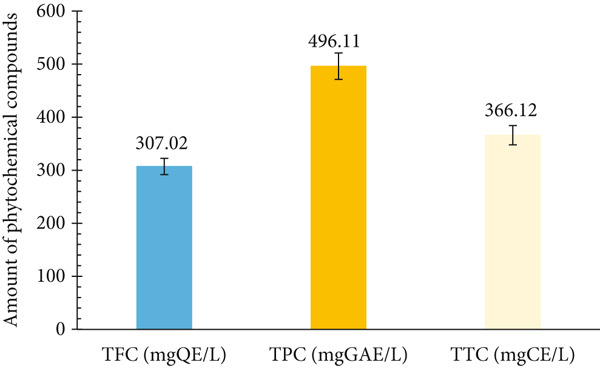
Phytochemical content of *foléré* beverage. TFC: total flavonoid content, TPC: total polyphenol content, TTC: total condensed tannin content, QE: quercetin equivalent, GAE: gallic acid equivalent, CE: catechin equivalent.

The TFC, TTC and TPC are presented in Figure 2. The TPC (496.11 ± 0.17 mgGE/L) was greater than the TTC (366.12 ± 0.09 mgCE/L) and TFC (307.02 ± 0.01 mgQE/L). The beverage is considered to be rich in polyphenols, followed by condensed tannins and lastly flavonoids. According to Ramirez‐Rodrigues et al. [43], the characterisation of hibiscus extracts revealed phenolic acids as the major bioactive compounds, with little or no flavonoid compounds. As such, it could be deduced that phenolic acids are the major bioactive compounds of hibiscus calyx. However, the polyphenol content in the current study is greater than those registered by Ramirez‐Rodrigues et al. [43] with the cold (286.58 and 279.13 mg/L) and hot (279.13 and 319.06 mg/L) extracts of fresh and dried hibiscus calyx. In the same line, Ajayi and Oyerinde [44] recorded a higher polyphenol content (31.2 ± 1.0 to 41.3 ± 0.39 mgGAE/g) with roselle tea enriched with ginger or lemon extracts. In contrast to our findings, Ahmed et al. [45] registered a high flavonoid content ranging between 134 and 250.5 mg/mL, followed by the polyphenol content ranging between 27.87 and 68.87 mg/mL and tannin content between 14.48 and 26.02 mg/mL, with the hot and cold extracts of hibiscus in Egypt. Comparatively, the TPC reported in this study was greater than 14.64 mg/mL registered by Bazán‐Plasencia et al. [46], but less than the total polyphenol and flavonoid contents registered by Sasikumar et al. [47] with citrus drink and nutraceutical beverage from blood fruit in Peru and India, respectively. Despite these differences, the hibiscus calyx can serve as a substitute to prepare the bioactive *foléré* especially during periods of scarcity of citrus fruits. The hibiscus calyx is more highly preserved than citrus fruits.

### 3.4. TAC and Carotenoid Content

The TAC and carotenoids content of *foléré* are presented in Table 3. *Foléré* is highly pigmented. As for the carotenoid content and derivatives, the lycopene content was greater than the *α*‐carotene, lutein and *β*‐carotene contents. The *α*‐carotene and *β*‐carotene contents were greater than the lutein content of the beverage. *α*‐Carotene and *β*‐carotene are principal precursors of pro–vitamin A, a vitamin needed to combat impaired vision. As such, regular consumption of *foléré* could help to combat impaired vision.

**Table 3:** Total monomeric anthocyanin content and carotenoid content of *foléré* beverage.

**Parameters**	**M** **e** **a** **n** ± **S** **D**
TAC (mg cyanidin‐3‐glycoside/L)	68.22 ± 1.07
*α*‐Carotene content (mg/100 mL)	29.58 ± 0.80
*β*‐Carotene content (mg/100 mL)	30.58 ± 1.85
Lycopene content (mg/100 mL)	18.84 ± 0.87
Lutein content (mg/100 mL)	71.63 ± 1.97

Abbreviations: SD, standard deviation; TAC, total monomeric anthocyanin content.

The TAC was greater than the carotenoid contents and derivatives. The high amount of TAC registered must have greatly contributed to the reddish colour of *foléré*. Anthocyanins are strong water‐soluble antioxidants and natural colourants of food with their attractive colours [48, 49]. However, the TAC registered in this study is less than those registered by Mgaya‐Kilima et al. [38] with roselle extract (555.3 mg/100 g) and roselle extracts blended with some tropical fruits (493.5–118.2 mg/100 g) but greater than those recorded by Omoarukhe [50] with unpasteurised (42.3 mg/L) and pasteurised (39.5 mg/L) roselle extracts. These differences are mostly related to the nature of the raw material, duration and temperature of extraction.

### 3.5. In Vitro Antioxidant Activities

The DPPH free radical scavenging activity, FRAP activity and ascorbic acid content of *foléré* are presented in Table 4. As for the antioxidant capacity of the beverage, 42.88% and 654.58 mgTE/L were registered for the DPPH and FRAP scavenging activities, respectively. The DPPH scavenging activity registered in this study is similar to the findings reported by Ahmed et al. [45] with roselle beverage in Egypt but lower than 65.72% with citrus drink reported by Bazán‐Plasencia et al. [46] in Peru. The FRAP activity was greater than 1.6 and 1.5 mmol/L reported by Mgaya‐Kilima et al. [38] with roselle beverage at ambient temperature or refrigerated in Tanzania but lower than those reported by Sasikumar et al. [47] with a nutraceutical beverage from blood fruit in India. These differences could be the result of the differences in the composition and production of the beverages. Indeed, antioxidant activities are highly correlated to the phenolic contents [12, 51]. Antioxidants exhibit protective functions in the body of living organisms. In this regard, *foléré* should not just be considered a drink taken to quench thirst but also a bioactive or functional beverage.

**Table 4:** Free radical antioxidant properties of *foléré* beverage.

**Parameters**	**M** **e** **a** **n** ± **S** **D**
FRAP (mgTE/L)	654.58 ± 0.36
DPPH (%)	42.88 ± 1.67
AAC (mg/100 mL)	6.5 ± 0.01

Abbreviations: AAC, ascorbic acid content; DPPH, 2,2‐diphenyl‐1‐picrylhydrazyl; FRAP, ferric reducing antioxidant power; SD, standard deviation; TE, Trolox equivalent.

Apart from phytochemical compounds, ascorbic acid is a strong antioxidant against free radicals [52]. An ascorbic acid content of 6.5 mg/100 mL was recorded for *foléré*. The ascorbic content was less than those reported by Gbadegesin et al. [36] and Fasoyiro et al. [37] with roselle drink blended with different tropical fruits (pineapple, orange and apple) in Nigeria. Similarly, Bazán‐Plasencia et al. [46] reported a higher ascorbic acid content with the nonalcoholic nutraceutical beverage from blood fruit in Peru. The technological production process and, more so, the type of raw materials must have accounted for these differences. According to Foundikou et al. [12], the technological process greatly affects the quality of the finished product.

### 3.6. Colour Intensity, Colour and Hue Tint Value

The colour intensity, colour and hue tint value of *foléré* are presented in Table 5. The colour intensity and hue tint value of 2.84 and 0.86 were recorded with the beverage. The colour intensity in the current study is greater than that registered by Gbadegesin et al. [36] with roselle drink fortified with pineapple juice extract and Bolade et al. [42] with the hot extracts of hibiscus calyx (0.088–0.218). At the same time, the colour intensity of the current study was less than 2.70 and 5.91 recorded by Ramirez‐Rodrigues et al. [43] and Ahmed et al. [45] with the hot and cold extracts of hibiscus. The differences in the colour intensity could be related to the technological process of extraction. Even if the present study showed a similar calyx/water ratio (1:40 w/v) to that reported by Ramirez‐Rodrigues et al. [43], the differences in the colour intensity could therefore be due to the temperature and the genealogy of the hibiscus species. The temperature and extraction time in the current study were set at 100°C/45 min and the pasteurisation at 65°C/30 min contrary to the temperature and extraction time of 90°C/16 min and 25°C/240 min for the hot and cold extracts of hibiscus reported by Ramirez‐Rodrigues et al. [43]. The colour intensity reduces with increasing temperature. This notion is supported by hue tint ratio. Hue tint measures the rate of colour degradation in anthocyanin‐containing products. Consequently, the higher the hue tint value, the higher the degradation of anthocyanin. Indeed, the tint value recorded in this study was greater than the values reported by Ramirez‐Rodrigues et al. [43] irrespective of the extraction procedure but falls within the range of 0.56–1 as reported by Ahmed et al. [45].

**Table 5:** Colour intensity, colour and hue tint value of *foléré* beverage.

**Parameters**	**M** **e** **a** **n** ± **S** **D**
Colour intensity	2.84 ± 0.09
Tint (T)	0.86 ± 0.07
Red colour (%)	61.40 ± 0.43
Yellow colour (%)	30.73 ± 0.11
Blue colour (%)	7.86 ± 0.54

Abbreviation: SD, standard deviation.

Furthermore, when the colour intensity was grouped into the visible spectrum, the percentage of red (61.40*%* ± 0.43*%*), yellow (30.73*%* ± 0.11*%*) and blue (7.86*%* ± 0.54*%*) colours was registered. The proportion of red colour is greater than those of yellow and blue. This must have accounted for the reddish‐purple colouration seen with the beverage. It could therefore be deduced that red is the predominant colour of *foléré*. This deduction is in line with the study conducted by Ramirez et al. [16] and Mgaya‐Kilima et al. [38] who described roselle beverage as being red.

### 3.7. Hygienic Quality of Foléré Beverage

The hygienic quality of *foléré* is presented in Table 6. *Foléré* presented an astonishing microbial load. All the hygienic indicators, TVCs, total fungi counts, total spore‐forming bacteria counts, total coliform counts and total faecal coliform counts remained within the acceptable limits. Despite the nondetection of total fungi, total coliforms and total faecal coliforms, the TVCs and total spore‐forming bacteria counts were 2 × 10^2^ and 1.5 × 10^2^ CFU/mL, respectively. These results were similar to those recorded by Bassey et al. [9] with roselle beverage fortified with extracts from garlic, ginger and moringa leaf powder after 48 h of storage. In this same perspective, Ukwo et al. [18] revealed a complete absence of the total coliform count with *zobo* enriched with date fruits, but the TVC in the current study exceeds the values recorded by the latter. On the contrary, Bayoï et al. [11] and Awe and Abdulmumini [53] registered a higher TVC and total fungi count with *foléré* and *zobo* enriched with tamarind leave extracts, lime and chemical preservatives. The differences in the microbiological qualities of *foléré* are closely related to the unhygienic sanitary environments, poor mastering of food processing techniques and the nature of tools and raw materials [5, 7].

**Table 6:** Hygienic quality of *foléré* beverage.

**Microbial flora**	**Microbial loads (CFU/mL)**	**Limit of satisfaction (CFU/mL)**
TVC	2 × 10^2^	> 10^6^
TSBC	1.5 × 10^2^	> 10^4^
TFCC	Nd	> 10^2^
TCC	Nd	> 10^3^
TFC	Nd	> 10^5^

Abbreviations: TCC, total coliform counts; TFC, total fungi counts; TFCC, total faecal coliform count; TSBC, total spore‐forming bacteria counts; TVC, total viable counts.

### 3.8. Bacteriological Characterisation

The molecular analysis of the sequenced amplicons using bioinformatics tools revealed that these organisms could possibly belong to the genus *Enterobacter* and *Aeromonas* (Table 7). *Enterobacter bugandensis* and *Aeromonas hydrophila* presented 89.47% and 71.8% similarity with the query sequences, respectively. The application of thermal treatments during the manufacturing of *foléré* could be considered inefficient to completely eliminate these microorganisms. According to Adebayo‐Tayo and Samuel [54] and Omemu et al. [55], the purple calyx of *Hibiscus sabdariffa* contains a wide variety of bacteria isolates. These bacteria may present some thermal resistance and consequently their presence in the finished product. Besides this, they could result from postproduction contaminations. *Enterobacter* and *Aeromonas* are food contaminants from diverse origins. Several research studies have reported the presence of *Bacillus subtilis*, *Pseudomonas* spp., *Staphylococcus aureus*, *Streptococcus faecalis*, *Escherichia coli*, *Proteus mirabilis*, *Serratia* spp. and *Veillonella* spp. with beverage samples in Nigeria and Ghana [55, 56]. The presence of these organisms is a call for concern.

**Table 7:** Bacteriological profile of *foléré* beverage.

**Genus**	**Species**	**Max score**	**Query cover**	**Percent identity**	**Accession number**
*Enterobacter*	*Enterobacter bugandensis*	1122	88%	89.47%	NR‐148649‐1
*Aeromonas*	*Aeromonas hydrophila*	342	96%	71.80%	NR‐119190‐1

## 4. Conclusion

*Foléré* is an acidic juicy reddish concoction of hibiscus calyx. The characterisation of *foléré* enriched with tamarind fruit pulp extract revealed the presence of reducing sugars, carbohydrates, protein, carotenoids and electrolytes. Similarly, the beverage contained polyphenols, flavonoids, anthocyanins, ascorbic acid and tannins, as well as important DPPH and FRAP scavenging activities. Hence, *foléré* can be considered a functional beverage. Furthermore, the hygienic quality is satisfactory and microbiologically acceptable, but the need to produce *foléré* that is less susceptible to microbial contamination is necessary to bridge the drawbacks surrounding its consumption.

## Conflicts of Interest

The authors declare no conflicts of interest.

## Funding

No funding was received for this manuscript.

## Data Availability

All the data supporting this study are included in the manuscript.
